# Role of Extracellular Vimentin in Cancer-Cell Functionality and Its Influence on Cell Monolayer Permeability Changes Induced by SARS-CoV-2 Receptor Binding Domain

**DOI:** 10.3390/ijms22147469

**Published:** 2021-07-12

**Authors:** Divyendu Goud Thalla, Philipp Jung, Markus Bischoff, Franziska Lautenschläger

**Affiliations:** 1 Leibniz Institute for New Materials, 66123 Saarbrücken, Germany; divyendu.thalla@uni-saarland.de; 2 Faculty of Natural Sciences and Technology, Experimental Physics, Saarland University, 66123 Saarbrücken, Germany; 3 Institute for Medical Microbiology and Hygiene, Saarland University, 66421 Homburg, Germany; Philipp.Jung@uks.eu (P.J.); markus.bischoff@uks.eu (M.B.); 4 Centre for Biophysics, Saarland University, 66123 Saarbrücken, Germany

**Keywords:** extracellular vimentin, IGF-1 receptor, cancer, SARS-CoV-2 receptor binding domain

## Abstract

The cytoskeletal protein vimentin is secreted under various physiological conditions. Extracellular vimentin exists primarily in two forms: attached to the outer cell surface and secreted into the extracellular space. While surface vimentin is involved in processes such as viral infections and cancer progression, secreted vimentin modulates inflammation through reduction of neutrophil infiltration, promotes bacterial elimination in activated macrophages, and supports axonal growth in astrocytes through activation of the IGF-1 receptor. This receptor is overexpressed in cancer cells, and its activation pathway has significant roles in general cellular functions. In this study, we investigated the functional role of extracellular vimentin in non-tumorigenic (MCF-10a) and cancer (MCF-7) cells through the evaluation of its effects on cell migration, proliferation, adhesion, and monolayer permeability. Upon treatment with extracellular recombinant vimentin, MCF-7 cells showed increased migration, proliferation, and adhesion, compared to MCF-10a cells. Further, MCF-7 monolayers showed reduced permeability, compared to MCF-10a monolayers. It has been shown that the receptor binding domain of SARS-CoV-2 spike protein can alter blood–brain barrier integrity. Surface vimentin also acts as a co-receptor between the SARS-CoV-2 spike protein and the cell-surface angiotensin-converting enzyme 2 receptor. Therefore, we also investigated the permeability of MCF-10a and MCF-7 monolayers upon treatment with extracellular recombinant vimentin, and its modulation of the SARS-CoV-2 receptor binding domain. These findings show that binding of extracellular recombinant vimentin to the cell surface enhances the permeability of both MCF-10a and MCF-7 monolayers. However, with SARS-CoV-2 receptor binding domain addition, this effect is lost with MCF-7 monolayers, as the extracellular vimentin binds directly to the viral domain. This defines an influence of extracellular vimentin in SARS-CoV-2 infections.

## 1. Introduction

Vimentin is a cytoskeletal filament of the family of intermediate filaments that has a vital role in cell migration, adhesion, and signaling due to its interactions with various proteins [1]. It forms a filamentous network that extends from the nuclear periphery to the plasma membrane. Vimentin is involved in cell physiology, inflammation, wound healing, and immune responses [2]. Its involvement in these cellular functions is believed to be through its dynamic phosphorylation. Interestingly, as well as being present in the cell cytoplasm, vimentin is found in the extracellular space around various cell types. Vimentin can thus be secreted into the extracellular space under various physiological conditions, such as cell activation, inflammation, senescence, and stress [3,4]. Vimentin is secreted by various cell types, such as macrophages, astrocytes, neutrophils, monocytes, apoptotic lymphocytes, and endothelial cells [3,5,6,7,8]. This extracellular vimentin can either remain bound to the cell surface (i.e., surface vimentin) or it can be secreted in an unbound form in the extracellular matrix (i.e., secreted vimentin).

In epithelium-derived cancers, intracellular vimentin has long been known to be involved in the process known as epithelial-to-mesenchymal transition, which describes a particular moment during the progression of tumor cells to metastases. Interestingly, surface vimentin has also been reported to be involved in this process, because cytoplasmic vimentin is translocated to the cell surface during epithelial-to-mesenchymal transition. This distinct property provides an opportunity to identify and isolate aggressive cancer cells, and thus to target them [9]. Surface vimentin has also been shown to have a role in other cancer cells, such as glioblastoma multiforme cancer stem cells, which are tumor-initiating cells that express vimentin on their surface. By targeting glioblastoma multiforme cells using an antibody against cell-surface vimentin (defined as the CSV antibody; clone 84-1), surface vimentin is internalized, which results in cell apoptosis and inhibition of tumor growth [10].

Surface vimentin also has a role in human circulating tumor cells (CTCs), with CTCs being detected using a CSV antibody. Surface vimentin has also been detected on neuroblastoma, osteosarcoma, and rhabdomyosarcoma cells [11]. In other studies, epithelial-to-mesenchymal transition induced CD45^−^ CTCs in patients with metastatic colorectal cancer, and CD133^−^ CTCs have been isolated from hepatocellular carcinoma using the CSV antibody [12,13]. Isolation of CSV-positive CTCs has resulted in quantification of metastatic cells and evaluation of cancer progression, which helps in clinical decision making.

Surface vimentin has been shown to be involved in the binding to cells and the internalization of numerous bacteria and viruses, such as DENV-2, *Listeria monocytogenes*, *Streptococcus pyogenes*, human Papillomavirus 16 [14,15,16,17]. It can also facilitate the entry of SARS-CoV by acting as a co-receptor between the SARS-CoV spike protein and the cell-surface angiotensin-converting enzyme 2 (ACE2) receptor [18]. Indeed, a recent study revealed that the binding of the SARS-CoV-2 spike protein to extracellular vimentin occurs during SARS-CoV-2 infection, and so vimentin was suggested to be a potential target for inhibition of viral particle binding to and entry into cells [19]. Furthermore, for cancer patients, immunosuppression is a major potential side effect, whereby immunosuppression increases the vulnerability of the patients to diseases caused by viral pathogens. Therefore, it is also vital to study the role of surface vimentin in viral and bacterial infections in the context of cancer progression.

To date, all of these studies have shown that detection of the levels of extracellular vimentin represents a diagnostic method and a therapeutic target in patients with sarcoma, and also allows analysis of metastatic precursor subpopulations. However, the functional role of extracellular vimentin in relation to cancer cells has not been studied. Thus, we aimed here to investigate the effects of vimentin on cancer cells in particular. From the literature, it is known that extracellular vimentin is a ligand for the surface-expressed pattern-recognition receptor dectin-1, natural cytotoxicity receptor 1 (NKp46), and insulin-like growth factor-1 receptor (IGF-1R) [20,21,22]. Of these, IGF-1R is overexpressed in cancer cells, has a crucial role in tissue development, and is activated by the hormone IGF-1. We therefore focused on the role of extracellular vimentin on IGF-1R, to which IGF-1 binds with high affinity [23]. This pathway has a vital role in cell-cycle progression, cell apoptosis, translation of proteins, and pathogenesis of autoimmune diseases [24].

**Figure 1:**
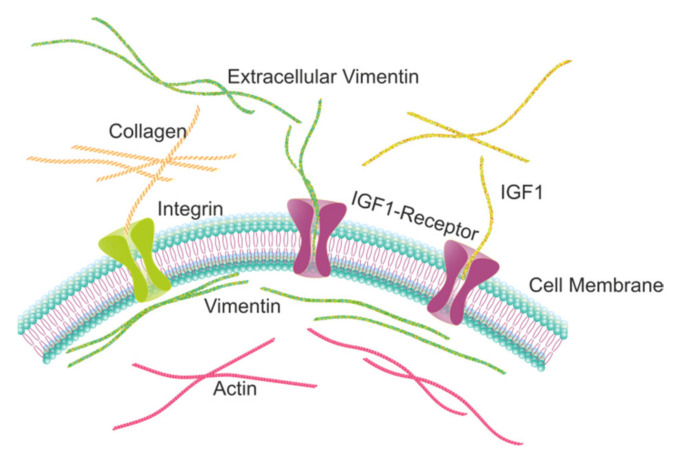
Illustration of the distributions of vimentin (**dark green**), actin (**violet**), and collagen (**light green**) fibers, and their associations with the cell membrane bilayer and the IGF-1 (**violet**) and integrin (**light green**) receptors.

A study on spinal cord injury in mice showed that extracellular vimentin can activate IGF-1R within the same signalling pathway as IGF-1 [22] (Figure 1). We thus hypothesized that extracellular vimentin has functional similarity to IGF-1 and can thus interact with cellular functions such as cell migration, proliferation, adhesion, and monolayer permeability. Here we studied these functions and extracted the relevant quantitative parameters (e.g., proliferation rate, migration speed, adhesion forces, and monolayer permeability) and compared them across non-tumorigenic (MCF-10a) and cancer (MCF-7) cells. This analysis showed that extracellular vimentin enhances these cellular functions and might thus be used to modulate them.

At the same time, currently in 2021, we are in the middle of a pandemic, and researchers around the world are looking for ways to interfere with the entry of SARS-CoV-2 into cells. Therefore, in the present study, we also investigated the role of extracellular vimentin in SARS-CoV-2 infection, particularly as viral shedding of coronavirus is greater in patients with cancers compared to those without [25]. A recent study showed the effects of SARS-CoV-2 spike proteins in terms of altered blood–brain barrier properties and integrity [26]. In addition to these effects on the throat and respiratory system, SARS-CoV-2 can have effects on multiple organ vasculatures, which can be lethal under certain circumstances [27].

The data reported here show that surface vimentin enhances the invasive potential caused by the SARS-CoV-2 receptor binding domain (RBD) of the spike protein in monolayers of both non-tumorigenic (MCF-10a) and cancer (MCF-7) cells. If, on the other hand, the RBD of SARS-CoV-2 spike protein binds to secreted extracellular vimentin, this would protect the integrity of the cell monolayers. As there is the need for ways to interfere with the entry of SARS-CoV-2 into human cells, this secreted extracellular vimentin might represent a valuable target for the modulation of SARS-CoV-2 entry into cells.

## 2. Results

These investigations were carried out using cells derived from human breast epithelium, as the control MCF-10a non-tumorigenic cells and the MCF-7 cancer cells, both of which express IGF-1R. The expression levels of IGF-1R are higher in MCF-7 cells compared to MCF-10a cells [28].

### 2.1. Extracellular Vimentin Promotes Proliferation in MCF-7 Cells through Activation of IGF-1R

To investigate the proliferation of MCF-10a and MCF-7 cells under the influence of extracellular vimentin, we carried out proliferation assays using 3-[4,5-dimethylthiazole-2-yl]-2,5-diphenyltetrazolium bromide (MTT). The cells were treated with increasing concentrations of recombinant human vimentin (0, 50, 100, 200 ng/mL) for 72 h. The absorbance was then measured using a plate reader, to define the viable cells.

**Figure 2:**
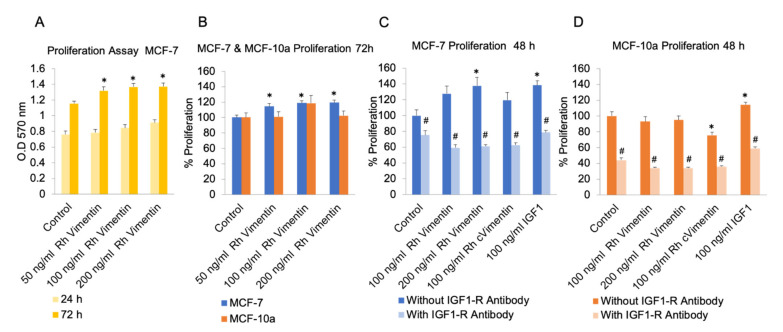
Proliferation of MCF-10a (control) and MCF-7 (cancer) cells under treatment with recombinant (Rh) vimentin. (**A**) Raw data for absorbance at 570 nm in the MTT assay for MCF-7 cells after 24 and 72 h. (**B**) Cell proliferation following 72 h treatments of Rh vimentin. (**C**,**D**) Proliferation upon blocking of IGF-1R with pretreatment with an anti-IGF-1R antibody along with Rh vimentin; IGF-1; Rh cVimentin (recombinant citrullinated form of vimentin). * *p* < 0.05, compared to control (unpaired *t*-tests). ^#^  *p* < 0.05, compared to corresponding counterparts without IGF-1R antibody (unpaired *t*-tests). Experiments were performed at least two times in triplicates. Error bars indicate SEM.

These data for the MCF-7 cells show that the absorbance, and therefore the cell metabolic activity, with addition of recombinant vimentin was greater than for the control cells (Figure 2B). At 100 ng/mL recombinant vimentin, MCF-7 cells showed increased proliferation rates, by ≥20%. For the MCF-10a cells, those treated with recombinant vimentin did not show significant change compared to untreated cells at both 48 and 72 h time points (Figure 2B,D).

It has been suggested in the literature that vimentin binds directly to and activates IGF-1R [22]. To determine whether or not cell proliferation was stimulated by a direct interaction between vimentin and IGF-1R, a 15-min pre-incubation with an anti-IGF-1R antibody (ARG51076; anti-IGF1 Receptor antibody; Arigobio) was used to block IGF-1R in the MCF-10a and MCF-7 cells, with cell proliferation monitored over 48 h (Figure 2C,D). Indeed, blocking IGF-1R inhibited 200 ng/mL vimentin-stimulated cell proliferation in MCF-7 cells. Here, we also treated cells with citrullinated vimentin, which is the form of vimentin that is secreted in patients suffering from rheumatoid arthritis, to see whether the effects seen here are different to those of recombinant vimentin. These data also indicated that addition of extracellular citrullinated vimentin did not improve proliferation in MCF-7 cancer cells through the activation of IGF-1R (Figure 2C). However, proliferation was significantly reduced in MCF-10a cells upon Rh cVimentin treatment (Figure 2D).

### 2.2. Extracellular Vimentin Promotes Stronger Adherence to the Underlying Substrate for MCF-7 Cells

**Figure 3:**
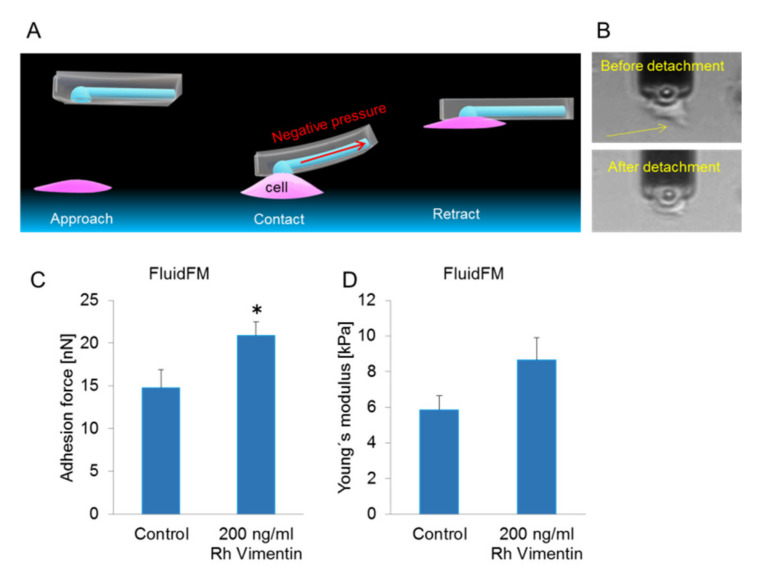
Adhesion force measurements of the MCF-7 (cancer) cells under treatment with recombinant (Rh) vimentin. (**A**) Illustration of the approach, with cell contact establishment and probe retraction during the FluidFM-based single-cell force spectroscopy. (**B**) Representative images showing an MCF-7 cell treated with 200 ng/mL recombinant vimentin which was detached by the retraction movement of the FluidFM probe. (**C**,**D**) Adhesion force (**C**) and stiffness (**D**) measured during the detachment of MCF-7 cells (n ≤ 7 cells per condition) after 24 h without and with 200 ng/mL vimentin. * *p* < 0.05, compared to control (0) (unpaired *t*-tests). Error bars indicate SEM.

As mesenchymal cell migration is strongly dependent on cell adhesion, we wanted to quantify the adhesion forces between these cells and their underlaying substrate. Therefore, we investigated the force necessary to detach the MCF-10a and MCF-7 cells from the fibronectin-coated glass substrate without and with the vimentin treatments, to quantify the adhesion strengths of the cells to this substrate. This was achieved using fluidic force microscopy (FluidFM). This is a particular single-cell force spectroscopy set-up with hollow cantilevers, which, in addition to the measurement of conventional cell mechanical properties, can be used to hold, immobilize, or move cells by negative pressure (Figure 3A,B) [29]. Unfortunately, the force spectroscopy measurements with the MCF-10a control cells led to cell disruption or disengagement of the attachment between the cells and micropipette, which thus overstrained the experimental set-up. Therefore, only the effects of vimentin on the adhesion forces of the MCF-7 cells were considered here. The untreated MCF-7 cells had a maximum detachment force of 14.7 ± 2.1 nN, while with 200 ng/mL vimentin treatment, this was significantly increased to 21.0 ± 1.5 nN (Figure 3C). This was also reflected in the modest increase (not statistically significant) to the young’s modulus of these cells upon vimentin treatment (Figure 3D). We also concluded that the adhesion force of the MCF-10a cells was particularly high and appeared to exceed the adhesion force of MCF-7 cells, although it cannot be quantified using this method.

Therefore, as indicated above, the vimentin-treated MCF-7 cells adhered more strongly to the substrate than the control MCF-7 cells.

### 2.3. Extracellular Vimentin Induces Migration of MCF-10a and MCF-7 Cells

**Figure 4:**
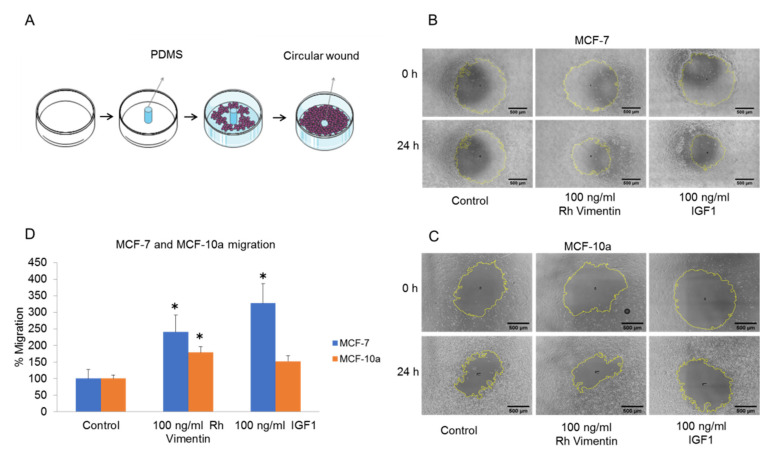
Gap closure assays for the MCF-10a and MCF-7 cells under treatment with recombinant (Rh) vimentin. (**A**) Schematic representation of the creation of the circular gaps using a PDMS column. (**B**,**C**) Representative images of the gap closure of MCF-10a cells (**B**) and MCF-7 cells (**C**) without and with treatments with vimentin and IGF-1 (scale bar 500 µm, yellow line indicates the edge of gap closure). (**D**) Migration rate is calculated by measuring gap closure (area covered by cell monolayer) over the time in terms of µm^2^/h and then it is normalized to control. To exclude effects of cell proliferation on cell migration, the cells were initially starved for 24 h in serum-free medium. * *p* < 0.05, compared to relevant control (unpaired *t*-tests). Error bars indicate SEM.

To further define the role of extracellular vimentin in the functions of these cells, its effects on cell migration were investigated using migration assays. To exclude the possibility that increased cell proliferation after vimentin treatment can obscure a migration result, the cells were initially serum-starved for 24 h. For the migration assays, a circular gap was prepared using soft polydimethylsiloxane (PDMS) pillars of 500 μm diameter on cell culture dishes (Figure 4A). Once the seeded cells had reached confluency, the PDMS pillars were removed, and cell migration was recorded over 24 h using video microscopy.

We analyzed the sizes of the gaps directly after removing the PDMS pillar using the ImageJ software, with calculation of the area (µm^2^) of gap closure per hour (Figure 4). Migration rate for MCF-10a cells treated with 100 ng/mL vimentin and 100 ng/mL IGF-1 were approximately 1.8 and 1.5 times higher compared to the untreated cells (Figure 4D). In the MCF-7 cancer cells, the migration rate upon 100 ng/mL vimentin and 100 ng/mL IGF-1 treatments, these reached 2.5 and 3 times higher than control respectively (Figure 4D). Gap closure was therefore faster after the treatment with extracellular vimentin in both the MCF-10a and MCF-7 cells. This suggests that activation of these cells with extracellular vimentin enhances cell migration, which results in faster closure of these wounds. Interestingly, the effects of added recombinant vimentin were again stronger in the MCF-7 cancer cells compared to the MCF-10a cells.

**Figure 5:**
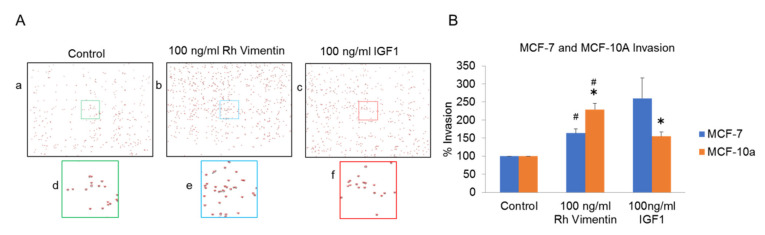
Transwell migration assays for the MCF-10a (control) and MCF-7 (cancer) cells under treatment with recombinant (Rh) vimentin. (**A**) Representative images used for the quantification of the MCF-10a cells that had migrated through the transwell membranes. (**B**) Quantification of the migrated cells without and with vimentin and IGF-1. Individual experiment was normalized to control and then mean values of three experiments were calculated. ^#^ * *p* < 0.05, compared to group indicating same symbol (unpaired *t*-tests). Error bars indicate SEM.

In addition to gap closure assay, we also carried out transwell migration assays. The MCF-10a and MCF-7 cells were placed in the upper reservoirs of individual systems and cultured in the presence or absence of Rh vimentin and IGF-1. The number of cells that moved through the porous membranes was then quantified at 48 h post-seeding (Figure 5).

Both the MCF-10a and MCF-7 cells showed enhanced migration through the porous membranes upon vimentin treatment, and for their adherence to the well plate, as compared to the untreated control cells (Figure 5). Interestingly, the IGF-1–treated MCF-7 cells showed higher migration rates when compared to the vimentin-treated cells, with the converse seen for the IGF-1–treated MCF-10a cells (Figure 5B). This effect complements our findings for gap closure (Figure 4D).

### 2.4. Extracellular Vimentin Effects on MCF-10a and MCF-7 Cell Monolayer Permeabilities and Alterations of Cell Monolayer Integrity Caused by the Receptor Binding Domain of SARS-CoV-2 Spike Protein

**Figure 6:**
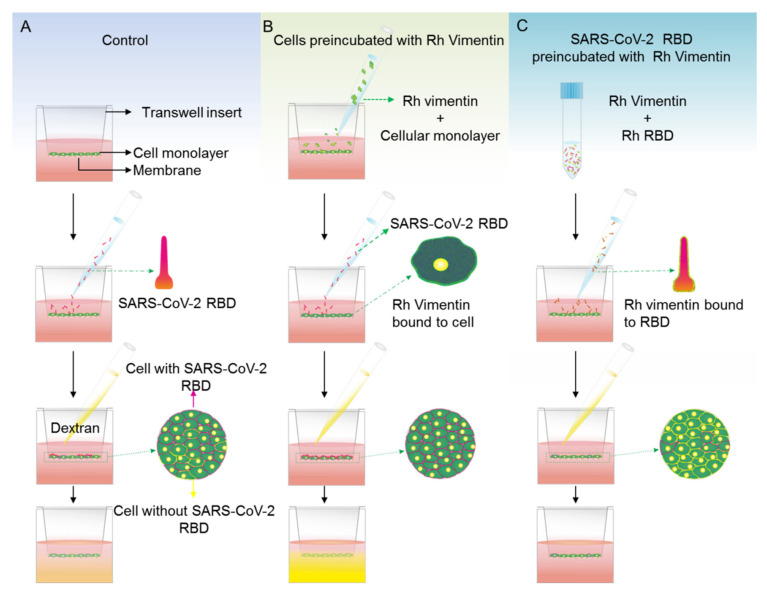
Stepwise procedures for determination of MCF-10a and MCF-7 cell monolayer permeabilities under SARS-CoV-2 RBD treatments using 3 kDa FITC-dextran. (**A**–**C**) Cell monolayers were treated for 24 h with SARS-CoV-2 RBD, either alone (**A**) or after treatment with recombinant (Rh) vimentin for 1 h (**B**), and with SARS-CoV-2 RBD that had been preincubated with Rh vimentin for 1 h (**C**).

Alterations to epithelial cell monolayer permeability are an indicator of disease conditions in epithelial tissues, as well as a marker of oncogenesis [30,31,32]. Such alterations might have a role in tumor invasiveness, and also in viral invasion into various organs. Indeed, it was shown in a recent study that SARS-CoV-2 receptor binding domain an affect the endothelial cell monolayer permeability [26]. Here, we measured the fluorescence intensity of 3 kDa FITC-dextran that passed through MCF-10a and MCF-7 cell monolayers upon addition of 10 nM recombinant vimentin and 10 nM SARS-CoV-2 RBD, using a protocol that is illustrated in Figure 6.

**Figure 7:**
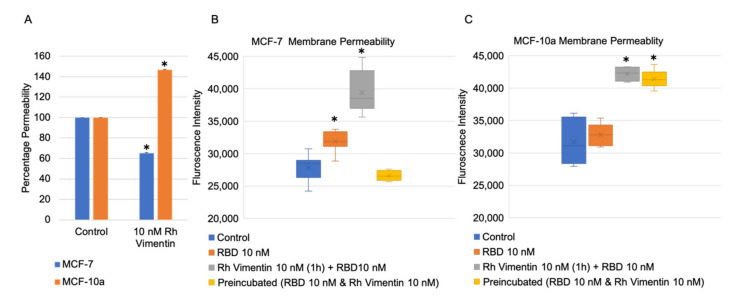
Monolayer permeabilities of MCF-10a and MCF-7 cells using 3 kDa of FITC-dextran treatment for 1 h. (**A**) Cell monolayers treated with 10 nM of recombinant (Rh) vimentin for 24 h, with data normalized to the controls. (**B**,**C**) Treatments of MCF-10a (**B**) and MCF-7 (**C**) cell monolayers without (control) and with SARS-CoV-2 RBD for 24 h (RBD), including 10 nM of vimentin monolayer pre-treatment for 1 h followed by SARS-CoV-2 RBD for 24 h; or 10 nM of vimentin and SARS-CoV-2 RBD pre-incubated together for 1 h, followed by SARS-CoV-2 RBD for 24 h. * *p* < 0.05, compared to control (unpaired *t*-tests).

First, the effects of recombinant vimentin on the permeability of MCF-10a and MCF-7 cell monolayers were determined. MCF-10a control cell monolayers treated with vimentin showed a 45% increase in the monolayer permeability compared to untreated MCF-10a control monolayers. Instead, monolayers of MCF-7 cells treated with recombinant vimentin showed the inverse trend, as a 35% decrease in monolayer permeability compared to the MCF-7 control monolayers (Figure 7A).

We then checked whether recombinant vimentin has an influence on MCF-10a and MCF-7 cell monolayer permeability when the monolayers are also exposed to SARS-CoV-2 RBD. Two different conditions for the recombinant vimentin treatment were used here. In the first (Figure 7B,C), the cell monolayers were pretreated with 10 nM of recombinant vimentin for 1 h prior to addition of 10 nM of SARS-CoV-2 RBD. After 24 h, for both MCF-10a and MCF-7 cells, their monolayer permeabilities were increased significantly compared to treatment of the monolayers with 10 nM of SARS-CoV-2 RBD alone. For the second condition, 10 nM of SARS-CoV-2 RBD and 10 nM of vimentin were preincubated together for 1 h, and then added to the cell monolayers. Interestingly, with the SARS-CoV-2 RBD and 10 nM of vimentin preincubation, the monolayer permeability alteration was not affected for the MCF-10a control cells, but it was inhibited for the MCF-7 cancer cells.

To summarize these data in general, we have shown that recombinant vimentin has effects on cell proliferation, adhesion, and migration, and on epithelial cell monolayer permeability in MCF-10a control cells and MCF-7 cancer cells. Vimentin also affects the cell monolayer permeability changes triggered by SARS-CoV-2 RBD.

## 3. Discussion

**Figure 8:**
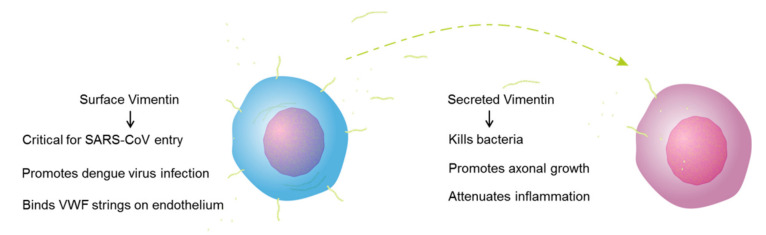
Functional roles of the different forms of extracellular vimentin, as surface vimentin and secreted vimentin. VWF, von Willebrand factor.

For about a decade, the existence of extracellular vimentin has been questioned by the scientific community, and even by researchers working on vimentin. However, recent studies have shown that vimentin can indeed be secreted into the extracellular space under several physiological conditions, such as cell activation, inflammation, senescence, and stress [3,4,33]. Vimentin is secreted by various cell types, such as macrophages, astrocytes, neutrophils, monocytes, apoptotic lymphocytes, and endothelial cells [3,5,6,7,8,33]. Mor–Vaknin et al. (2003) reported that activated macrophages secrete vimentin when treated with okadaic acid. Recently, it was shown that oxidized low-density lipoprotein induced vimentin secretion via CD36 in macrophages [34]. Recent studies have also provided evidence that extracellular vimentin is involved in several diseases, in repair mechanisms for spinal cord injury, and in the infection mechanisms of viruses [35]. We also described some of these roles of extracellular vimentin in health and disease in a recent review [2]. The functions of extracellular vimentin are still under debate, however, and these functions appear to depend on the form of this extracellular vimentin (Figure 8).

Secreted vimentin could have a potential role in wound healing, although to date, the role of vimentin in wound healing has mainly focussed on cytoplasmic vimentin. Nevertheless, recent studies have explored the possibility of extracellular vimentin as a potential remedy for tissue repair in many injuries [2]. Post-injury, vimentin released into the extracellular milieu facilitates wound closure by binding to mesenchymal leader cells. This extracellular vimentin promotes mesenchymal to myofibroblast differentiation of leader cells [36]. However, here the interaction of extracellular vimentin with receptors on leader cells that lead to wound healing remains an open question.

Vimentin has also been shown to have a role in wound healing during functional recovery after trauma in the central nervous system, which is a challenge that is still faced in the field of neuroscience [37]. In this context, a newly described compound, denosomin, was shown to provide added benefits in the treatment of mice for spinal cord injury [38]. During the course of these treatments, it was noted that the astrocytes tended to secrete vimentin at the site of the injury. This secreted vimentin promoted axonal growth by activation of IGF-1R, to thus promote improved functional recovery of the spinal cord in mice [22]. However, this previous study was focused mainly on the promotion of axonal growth via the vimentin interaction with IGF-1R, so in the present study we specifically explored the role of the extracellular vimentin and IGF-1R interaction across a range of cellular functions, as cell proliferation, adhesion, and migration; these are all crucial for tumor progression.

In cancers, the collective migration of cells is a critical event for establishment of metastases, and this indicates how cells contribute to cancer invasion [39]. Extracellular vimentin was shown previously to be involved in cancer-cell invasion [40]. In another study, the interaction between surface vimentin and GlcNAc-polymers led to an increase in migration and invasion of MDCK and MCF-7 cells [41]. In the present study, we showed that addition of recombinant vimentin promoted wound closure for both MCF-10a and MCF-7 cells, presumably through increased migratory speed of the cells following activation of the IGF-1R cell-surface receptor. We also showed that recombinant vimentin stimulated a greater increase in cell migration rate in the MCF-7 cancer cells than for the MCF-10a cells. Here, we also compared the effect of vimentin on cell migration with that of IGF-1 itself, as it is well known that IGF-1 induces cell migration in breast epithelial cells [42]. Interestingly, in the MCF-10a cells, the addition of vimentin resulted in greater migratory speed for these cells compared to the addition of IGF-1. We also saw similar effects by using the transwell migration assay here, which was greater with treatment with vimentin, and even higher than for the IGF-1 treatment for the MCF-10a cells.

In invasion by cancer cells, cell adhesion is one of the first steps during metastasis [43]. In addition, cell migration and proliferation are regulated by cell adhesion to the extracellular matrix [44]. Therefore, we additionally investigated cell adhesion upon vimentin treatment in the present study, which showed that vimentin treatment increased the adhesion strength in MCF-7 cancer cells. Interestingly, we also showed that the vimentin-treated MCF-7 cells showed a higher trend towards Young’s modulus during these FluidFM measurements. This suggests that the stiffness of the MCF-7 cells was increased by vimentin.

Higher circulating IGF-1 levels are indicative of higher risk of breast cancer in premenopausal women [45,46], and it has been reported that IGF-1 stimulates proliferation of breast cancer cells [47,48]. Although serum vimentin expression has been reported for various cancers, these previous studies did not emphasize the significant role of extracellular vimentin in cell proliferation [49,50]. Therefore, we also tested whether this extracellular (soluble) vimentin had similar effects as IGF-1 on cell proliferation. Interestingly, treatment with vimentin promoted increased proliferation rates in the MCF-7 cancer cells but not in MCF-10a cells. This effect was diminished when IGF-1R was blocked (using an anti-IGF-1R antibody), which further supports our hypothesis that vimentin binding to IGF-1R is involved in general cellular functions.

Conclusively, as the migration rate of the MCF-10a cancer cells was higher in the presence of vimentin than the migration rate of MCF-7 cells in transwell assays under the same conditions, we hypothesized that transwell migration assays in the presence of vimentin can be used for sorting cancer cells. From this study, we concluded that cancer cells tend to be more sensitive to extracellular vimentin, and hence that extracellular vimentin has an effect on general cellular functions. Taking these parameters into consideration during clinical decision making can have a major role in the treatment of patients with cancers in general, and also under specific disease conditions.

**Figure 9:**
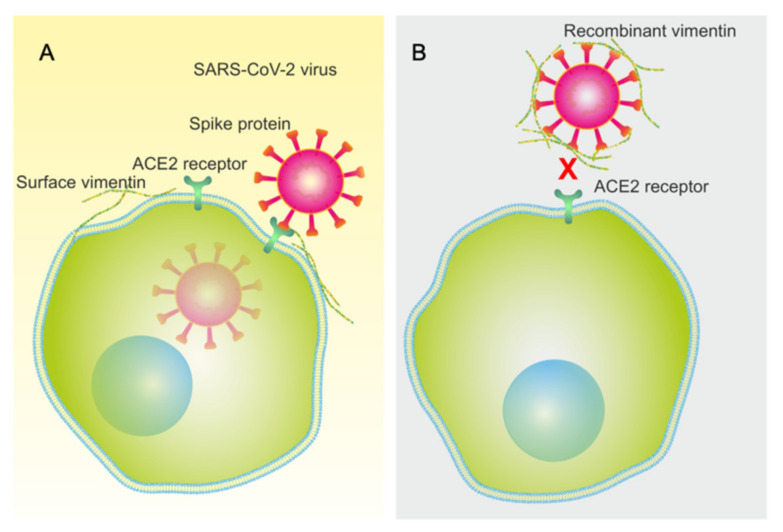
Illustration of the influence of extracellular vimentin in SARS-CoV-2 viral particle entry into cells. (**A**) Vimentin on the cell surface can act as a co-receptor, to promote further viral membrane association and virus entry into the cells. (**B**) For the viral particles pre-treated with recombinant vimentin, this instead restricts viral association with the cell membrane, and thus virus entry into the cells.

Extracellular vimentin has a vital role in various viral and bacterial infections [35]. A recent study that used a pseudo virus showed that surface vimentin is involved in SARS-CoV-2 infection, through its binding by the viral spike protein. Using an antibody against extracellular vimentin, they showed that vimentin can be used as a potential target to inhibit viral particle entry into cells [19]. In the present study, in the cell permeability assays, vimentin decreased the barrier permeability in MCF-7 cancer cells and increased it in MCF-10a cells. These MCF-7 cancer cells overexpress IGF-1R, which might lead to more binding of vimentin to the cell membrane, and a block (i.e., decreased permeability) of the paracellular junctions. The permeability increases in the cell monolayers of both MCF-10a and MCF-7 cells induced by SARS-CoV-2 RBD and was further enhanced when the cells were pretreated with vimentin. This effect might be because extracellular vimentin can act as a co-receptor for the SARS-CoV and SARS-CoV-2 spike proteins [18]. This role for vimentin as a co-receptor will lead to more attachment of the SARS-CoV2 RBD to the cells treated with vimentin, and will also affect the cell monolayer permeability. This might result in enhanced viral particle invasion into tissues and internalization into cells (Figure 9A).

In a previous study, pre-incubation of viral particles with recombinant vimentin restricted human Papillomavirus 16 (HPV) viral entry into Hela, HaCaT, and NIKS cells [17]. In the present study, we used the same method, and preincubated SARS-CoV-2 RBD and recombinant vimentin prior to the treatment of the cell monolayers with this mixture. In the MCF-7 cancer cells, the permeability was decreased by SARS-CoV-2 RBD. However, this effect was not seen for the MCF-10a cells. This could be due to lack of ACE2 receptor, that RBD does not have any additional effect on MCF-10a cells [51].

Further studies are required to understand the full mechanisms involved in the phenomena described here. However, we have shown that extracellular vimentin influences a range of cellular functions and might become an important player in the treatment of diseases or the prevention of particular infections. Additionally, extracellular vimentin can exist in different isoforms such as oxidized vimentin, citrullinated vimentin, and carbamylated vimentin which undergo post-translational modifications under certain circumstances (senescence, rheumatoid arthritis) [4,52,53]. Imitation of recombinant vimentin as extracellular vimentin could pose some limitations in the practical setting. Therefore, future studies should investigate the effect of native isoforms of extracellular vimentin on cells.

## 4. Materials and Methods

### 4.1. Cell Culture

The MCF-10A spontaneously immortalized breast epithelial (non-tumorigenic) cells were a kind gift from Marc Stemmler (FAU-Erlangen, Germany), and were cultured in Dulbecco’s modified Eagle’s medium/F12 supplemented (Gibco, Bleiswijk, The Netherlands) with 5% horse serum (Fisher Scientific, Schwerte, Germany), 5% penicillin/streptomycin (Gibco, NY, USA), 20 ng/mL of epidermal growth factor (PeproTech, Hamburg, Germany), 0.5 μg/mL of hydrocortisone (Sigma Aldrich, MO, USA), 100 ng/mL of cholera toxin, (Sigma Aldrich, MO, USA), 10 μg/mL of insulin (Sigma Aldrich, MO, USA), and 10 mM of HEPES (Gibco, Bleiswijk, The Netherlands). The MCF-7 malignant breast epithelial (cancer) cells were cultured in Dulbecco’s modified Eagle’s medium/F12 supplemented with 10% fetal bovine serum (Fisher Scientific, Schwerte, Germany), 5% penicillin/streptomycin (Gibco, NY, USA), and 5% Glutamax (Gibco, Paisley, UK).

### 4.2. Proliferation Assay

The MTT assay was used to monitor cell proliferation. Upon treating cells with MTT, the viable cells reduce the MTT reagent to formazan, with the formation of purple crystals. These crystals were dissolved by incubation of the samples in dimethylsulfoxide. The numbers of viable cells were proportional to the absorbance.

The MCF-10A and MCF-7 cells were plated into 96-well plates at 10,000 cells/well and were left to attach for 24 h. The medium was then replaced with serum-free medium, for starvation of the cells for 12 h. The cells were treated with various concentrations of recombinant vimentin (R&D systems, Minneapolis, MN, USA), citrullinated vimentin (Cayman Chemical, Ann Arbor, MI, USA), and/or recombinant IGF-1 (Invitrogen, Waltham, MA, USA) in serum-free medium for 24 h, as indicated. After aspiration of the medium, the cells were incubated in fresh medium with fetal bovine serum for 24 and 72 h. The cells were then washed with phosphate-buffered saline (PBS) and treated with 0.5 mg/mL MTT (Sigma Aldrich, St. Louis, MO, USA) for 3 h. Then 100 µL of dimethylsulfoxide was added to each well to dissolve the purple formazan crystals that had formed. After 15 min, the absorbance was measured at 570 nm in a plate reader (Infinite M200 Pro; Tecan, Crailsheim, Germany). For blocking the IGF-1 receptor, the cells were pre-incubated with 1.5 μg/mL anti-IGF-1R antibody (ARG51076; Arigo bio, Hsinchu, Taiwan) for 15 min and then incubated with recombinant vimentin, citrullinated vimentin, and IGF-1 for 48 h.

### 4.3. Migration Assay

Soft PDMS chips were made by mixing a curing agent and PDMS base (1:30). After curing in an oven at 75 °C for 1 h, pillars of 500 µm diameter were cut using a PDMS punch. These PDMS pillars were attached to each well in an eight-well chambered coverslip (Ibidi µ-slide, Gräfelfing, Germany). Then the MCF-10a and MCF-7 cells were seeded into each (50,000 cells/well) and incubated until confluent monolayers had formed. The cells were starved without serum for 24 h, and then the medium was replaced with fresh serum-free medium containing various concentrations of recombinant vimentin and IGF-1, as required. After 24 h, the medium was replaced with fresh medium (with fetal bovine serum). The PDMS pillars were then removed, which left a circular wound in each well, and the wound closure was monitored and recorded under an inverted microscope (Ti-Eclipse; Nikon, Düsseldorf, Germany) up to 24 h. During the imaging, the temperature in the chamber (Okolab, Ambridge, PA, USA) was maintained at 37 °C, and also using 5% CO_2_.

### 4.4. Transwell Migration Assay

Transwell inserts (pore size, 8 µm; Corning, Corning, NY, USA) were used for the MCF-10A and MCF-7 cell migration experiments. Inserts were placed in 12-well plates and 200 µL of cell solution was loaded into the upper chamber for 24 h, to attach to the membrane. The cells attached to the membrane were then starved without serum for 12 h. The medium in the upper chamber was replaced with fresh medium containing 100 ng/mL IGF-1 and 100 ng/mL recombinant vimentin. Then, 500 µL medium with serum was loaded into the bottom chamber. The transwell chambers were incubated for 48 h at 37 °C, to allow migration of the cells from the upper chamber into the lower chamber. Furthermore, the loading side of the upper chamber was cleaned using a cotton swab, to remove the cells that did not invade. Fresh medium containing 250 ng/mL Hoechst (Sigma Aldrich, MO, USA) was added to the bottom chamber, and then the upper chamber was placed into it for 20 min to stain the nuclei of the cells that had invaded the lower side of the membrane. Before imaging of the cells, the medium containing Hoechst was replaced with fresh medium.

### 4.5. Single-Cell Force Spectroscopy

Single-cell force spectroscopy was performed on an atomic force microscope (Flex-Bio) in FluidFM mode (Nanosurf GmbH, Liestal, Switzerland) and with FluidFM hollow micropipettes (Cytosurge, Glattburg, Switzerland), with a spring constant of 0.3 N/m and an opening diameter of 4 µm. The experiments were carried out with a relative force trigger of 8 nN and a z-range of 30 µm. Prior to the experiments, glass-bottomed dishes were coated with 25 µg/mL fibronectin (Sigma Aldrich, MO, USA) for 1 h. Subsequently, the cells (1 × 10^6^ cells/dish) were seeded into the glass-bottomed dish and allowed to attach for 24 h. Next, the cells were treated with 200 ng/mL recombinant vimentin and/or 100 ng/mL IGF-1 for 24 h. The cells were approached with the FluidFM micropipette, grabbed on the apical top, held by negative pressure (−500 mbar), and detached from the coated glass substrate by the retraction movement of the micropipette. Experimental data were only obtainable for the MCF-7 cells, as this procedure led to cell disruption or disengagement of the attachment between the cells and the spectroscopy probe. Thus, the forces were recorded only for the MCF-7 cells, as force–distance curves. Successful cell detachment was monitored by light microscopy (Zeiss AG, Oberkochen, Germany). The force–distance curve analysis was carried out with the SPIP software, version 6.6.2 (Image Metrology, Hørsholm, Denmark).

### 4.6. Permeabilty Assay

Transwell inserts (pore size, 0.4 µm; Corning, ME, USA) were used for the permeability assays for both the MCF-10A and MCF-7 cells. The inserts were placed into 12-well plates, and 200 µL of cells was loaded into the upper chamber (5000 cells/well). The cells were left to attach and form monolayers. The cell monolayers were then treated with 10 nM of recombinant vimentin. For the SARS-CoV-2 RBD analysis, the samples were treated under four primary conditions: (i) control cells as only cell monolayers; (ii) cell monolayers treated with 10 nM of SARS-CoV-2 RBD (R&D systems, MN, USA) for 24 h; (iii) cell monolayers treated with 10 nM of recombinant vimentin for 1 h and then with the addition of 10 nM of SARS-CoV-2 RBD for 24 h; and (iv) 10 nM of Rh vimentin and 10 nM of SARS-CoV-2 RBD were preincubated together for 1 h, and then added to cell monolayers for 24 h. After the appropriate treatments, 1 mg/mL of 3 kDa FITC-dextran (Sigma Aldrich, MO, USA) was added to the chambers and incubated for 1 h. The fluorescence intensities for the basolateral medium were then determined using a plate reader (Infinite M200 Pro; Tecan, Crailsheim, Germany).

## Data Availability

Not applicable.

## Abbreviations

ACE2	Angiotensin-converting enzyme 2
BSA	Bovine serum albumin
CSV	Cell-surface vimentin
CTCs	Circulating tumor cells
FluidFM	Fluidic force microscopy
IGF-1	Insulin-like growth factor-1
IGF-1R	Insulin-like growth factor-1 receptor
MCF-7 cells	Human breast cancer epithelial cell line
MCF-10a	Non-tumorigenic epithelial cell line
MTT	3-[4,5-dimethylthiazole-2-yl]-2,5-diphenyltetrazolium bromide
PBS	Phosphate-buffered saline
PDMS	Polydimethylsiloxane
RBD	Receptor binding domain
SARS-CoV	Severe acute respiratory syndrome coronavirus
